# Sequence and intramolecular distance scoring analyses of microbial rhodopsins

**DOI:** 10.12688/f1000research.7920.2

**Published:** 2016-04-06

**Authors:** Miki Asano, Shunta Ide, Atsushi Kamata, Kiyohiro Takahasi, Tetsuji Okada

**Affiliations:** 1 Department of Life Science, Gakushuin University, Tokyo, Japan

**Keywords:** Membrane, receptor, opsin, crystallography, coordinates

## Abstract

Recent accumulation of sequence and structural data, in conjunction with systematical classification into a set of families, has significantly advanced our understanding of diverse and specific protein functions. Analysis and interpretation of protein family data requires comprehensive sequence and structural alignments. Here, we present a simple scheme for analyzing a set of experimental structures of a given protein or family of proteins, using microbial rhodopsins as an example. For a data set comprised of around a dozen highly similar structures to each other (overall pairwise root-mean-squared deviation < 2.3 Å), intramolecular distance scoring analysis yielded valuable information with respect to structural properties, such as differences in the relative variability of transmembrane helices. Furthermore, a comparison with recent results for G protein-coupled receptors demonstrates how the results of the present analysis can be interpreted and effectively utilized for structural characterization of diverse protein families in general.

## Introduction

Microbial rhodopsins (MRs) are retinal proteins found in archaea, bacteria and eukaryotic algae. They share a common architecture including a heptahelical transmembrane (7TM) bundle and function as either light-dependent proton/ion transporters or photon sensors. Recent introduction of these proteins to brain research has substantially advanced our understanding of neuronal functions ^1, 2^. As a prototypical member of this family, bacteriorhodopsin (bR) and its proton-pumping mechanism have been studied extensively over the past forty years ^3, 4^. There are more than 130 wild type and mutant crystal structure entries of bR deposited in the Protein Data Bank (PDB). In addition to other retinal proteins found early in archaea, such as halorhodopsins and sensory rhodopsins, recent studies have demonstrated the presence of a number of proteins belonging to MR family in a wide range of organisms ^5, 6^. Crystal structures obtained for some of these proteins have shown that the arrangement of the seven helices is conserved ^7, 8^, and their 7TM domains are valuable for examinations of the effects of experimental conditions and sequence variation on structure.

Another class of well-known 7TM proteins are G protein-coupled receptors (GPCRs), for which more than 120 crystal structure entries are available in PDB. They all activate heterotrimeric G proteins upon agonist binding, but their seven helices exhibit significant divergence, reflecting a high degree of ligand variation, from small amines to peptide hormones ^9, 10^. A recent study demonstrated that, despite such variation among GPCRs, some conserved features in terms of intramolecular atomic distances were discernible ^11^. This observation was based on a systematic analysis of C _α_−C _α_ distances in crystal structures archived in PDB and hereafter we refer to this method as distance scoring analysis (DSA). For DSA, scoring of distance conservation among a set of crystal structures is simply made by taking the inverse of the coefficient of variation, wherein this coefficient is the average divided by the standard deviation.

If the number of available structures for this analysis was enough, it would be expected that structural differences due to either experimental conditions or sequence variation could be separately evaluated. In the previous analysis on GPCRs, we mainly focused on how the scores for C _α_−C _α_ distances in the 7TM bundle change as more variation in sequence was included because the apparent structural differences among receptors from different classes (rhodopsin-like, and others) were so large ^11^.

In the present study, we show that the DSA approach previously applied to GPCRs is also useful for highlighting bR and other MR helical regions that are relatively insensitive to the factors possibly affecting 7TM bundle structures. From the analysis of wild-type dark-state bR structures, we found that crystal packing could affect variability of a specific region of the 7TM bundle. On the other hand, the analysis of all MRs of known structure suggests that the regions involving high-score C _α_−C _α_ distances appear to be highly correlated with the functional importance. Furthermore, a comparison between two classes of 7TM proteins, MRs and GPCRs, demonstrates how the present analysis can be applied to diverse proteins families in general.

## Results

Raw data for DSA (Figure 2–Figure 6, Figure S3–Figure S6) Click here for additional data file. Copyright: © 2016 Asano M et al. 2016 Data associated with the article are available under the terms of the Creative Commons Zero "No rights reserved" data waiver (CC0 1.0 Public domain dedication).

Python script for making a score vs distance plot Click here for additional data file. Copyright: © 2016 Asano M et al. 2016 Data associated with the article are available under the terms of the Creative Commons Zero "No rights reserved" data waiver (CC0 1.0 Public domain dedication).

### Sequence analysis

Aside from the conventional serial numbering of polypeptide amino acids from the amino terminus, a common numbering system for a set of proteins based on conserved positions is expected to facilitate comparative protein family studies. A remarkable example involves GPCRs, for which an amino acid position in 7TM helices is given a common number (a BW number) ^12^. For example, the most highly conserved asparagine in helix I is referred to as 1.50 and the other residues in the helix are numbered in descending order toward the amino-terminal side or increasing order toward the carboxyl-terminal side. Thus, our selection of helix I in the previous analysis corresponds to a polypeptide range of 1.35 to 1.59 (25 residues). Such a clear definition of polypeptide positions is very important for the quantitative analysis of structures that have different underlying sequences.

**Figure S1.**
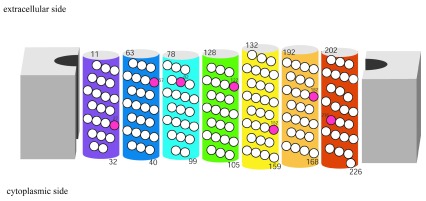
Schematic drawing of the 7TM bundle of MRs showing the proposed position of the *.50 residue in each helix (colored pink). The numbers attached to the helices are the selected ranges and the positions in the bR sequence.

Since no such numbering scheme has been proposed for MRs, we first analyzed the amino acid sequences for this family archived in the InterPro database ( www.ebi.ac.uk/interpro/) and identified the most conserved position in each of 7TM helices. Based on 603 sequences that include archaeal (178), bacterial (182), and algal (243) retinal proteins, an alignment was created and the distribution of amino acid types at each position was obtained, as shown in Table S1. The most highly conserved residue position in each helix was identified ( Figure S1) and assigned a number, *.50, in which “*” indicates a letter for helix identification. Since helix assignment with A to G has been frequently used for MRs, we follow this convention for helix description of this family. However, for residue numbering, we use numerals 1 to 7 for “*” in the present study in order to avoid confusion with single letter representation of amino acids.

**Figure S2.**
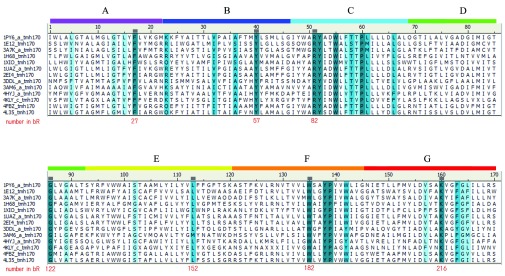
Sequence alignment of the 7TM regions of 13 MRs in set 3. The conserved positions are colored as follows: dark blue, identical; blue, strong similarity; light blue, weak similarity, according to PAM250 matrix definition. The small squares on the ruler indicate the *.50 positions of the numbering proposed for MRs.

Since we only considered possible retinal proteins, the amino acid type at 7.50 was Lys, and it exhibited 100% conservation ( Table 1). Helix F contained three highly conserved residues, which we designated 6.50 (Trp), 6.53 (Tyr), and 6.54 (Pro). The degree of conservation was very similar for 6.50 and 6.53, and higher than 97% among the 603 sequences. Helix C also contained a set of positions that exhibited greater than 95% conservation. At all these positions, the amino acid types, except 5.50 in helix E, were identical among the 13 MRs examined by DSA in the present study ( Figure S2).

**Table 1.** The 7TM bundle of 170 residues used for the present study and the proposed common numbering for microbial retinal proteins.

Helix	Common numbering	*.50 in bR	*.50 conservation (%)	Numbering in bR	Helix length
A	1.34–1.55	Phe27	64	11–32	22
B	2.33–2.56	Tyr57	95	40–63	24
C	3.46–3.67	Arg82	98	78–99	22
D	4.33–4.56	Gly122	95	105–128	24
E	5.30–5.57	Leu152	70	132–159	28
F	6.36–6.60	Trp182	98	168–192	25
G	7.36–7.60	Lys216	100	202–226	25

### Data selection

**Figure 1.**
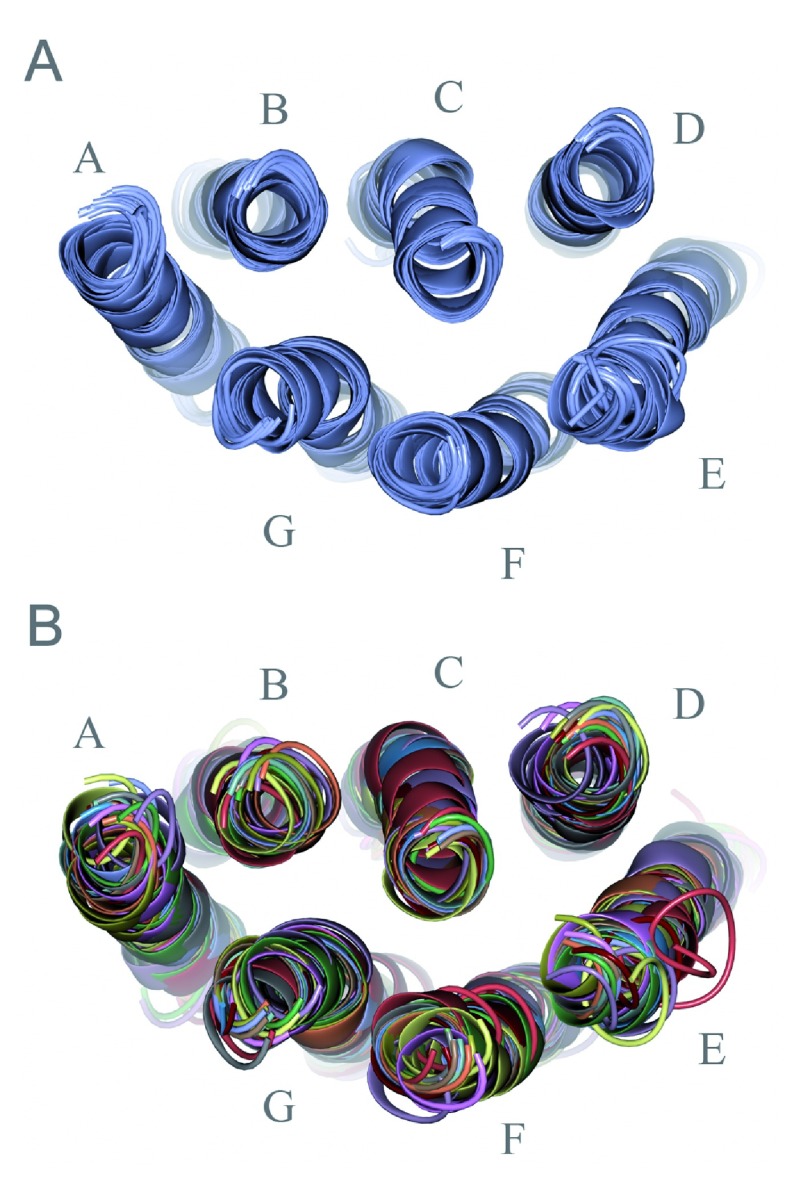
Graphical representation of MRs with known structure. **A**. 22 polypeptide chains in set 2 (dark-state wild-type bR structures) and **B**. 13 unique chains in set 3 (MRs of different sequences).

As of February 5_2015, there were 135 entries for MRs in PDB and the contents are summarized on our website ( www.gses.jp), which does not include redundant or outdated structures. By examining the superimposed chains from various proteins, we selected a range of amino acids for each of the seven helices with at least 22 residues per helix (~ 6 turns for regular geometry), resulting in a total of 170 residue bundles. Thus, we considered 14,365 C _α_−C _α_ pairs per 7TM bundle for the present analysis. From this archive, we made several data sets containing different combinations of polypeptide chains. Set 1 consisted of 9 chains of wild-type, dark-state bR, each of which represents a structure solved in a distinct space group or by a different research group. Set 2 ( Figure 1A) was more redundant than set 1, including multiple chains per entry, resulting in a total of 22 chains ( Table S2). Set 3 contained a set of 13 chains ( Figure 1B), each from structures with a unique sequence, as shown in Table 2. The other sets included, for instance, bR mutants, dark-state halorhodopsins and sensory rhodopsins. The results for these sets, other than 1 to 3, will be described elsewhere (Ono *et al.* unpublished report).

**Table 2.** The crystallographic models used for set 3 in the present study.

PDB ID	Protein	Species	Domain
1PY6	Bacteriorhodopsin	*H. salinarum*	archaea
1E12	Halorhodopsin	*H. salinarum*	archaea
3A7K	Halorhodopsin	*N. pharaonis*	archaea
1H68	Sensory Rhodopsin II	*N. pharaonis*	archaea
1XIO	Anabaena Sensory Rhodopsin	Nostoc sp. PCC 7120	bacteria
1UAZ	Archaerhodopsin-1	*H. chaoviator*	archaea
2EI4	Archaerhodopsin-2	*H. chaoviator*	archaea
3DDL	Xanthorhodopsin	*S. ruber*	bacteria
3AM6	Acetabularia Rhodopsin II	*A. acetabulum*	eukaryota
4HYJ	Proteorhodopsin (green)	*E. sibiricum*	bacteria
4KLY	Proteorhodopsin (blue)	HOT75	bacteria
4FBZ	Deltarhodopsin-3	*H. thermotolerans*	archaea
4L35	Cruxrhodopsin-3	*H. vallismortis*	archaea

### Distance analysis for bR structures

In all PDB entries for MRs, the most abundant structure was bacteriorhodopsin from *Halobacterium salinarum*. Thus, we are interested in determining how effective DSA is in detecting the intramolecular structural conservation among the ground-state wild-type bRs. The superimposed projection view of 22 chains in set 2 is shown in Figure 1A. These are obviously very similar to each other and are within the overall pairwise root-mean-squared deviation of 1.2 Å for 170 C _α_ positions ( Table S4). This similarity corresponds to a pairwise correlation coefficient of more than 0.993 calculated for the 14,365 C _α_−C _α_ distances.

**Figure 2.**
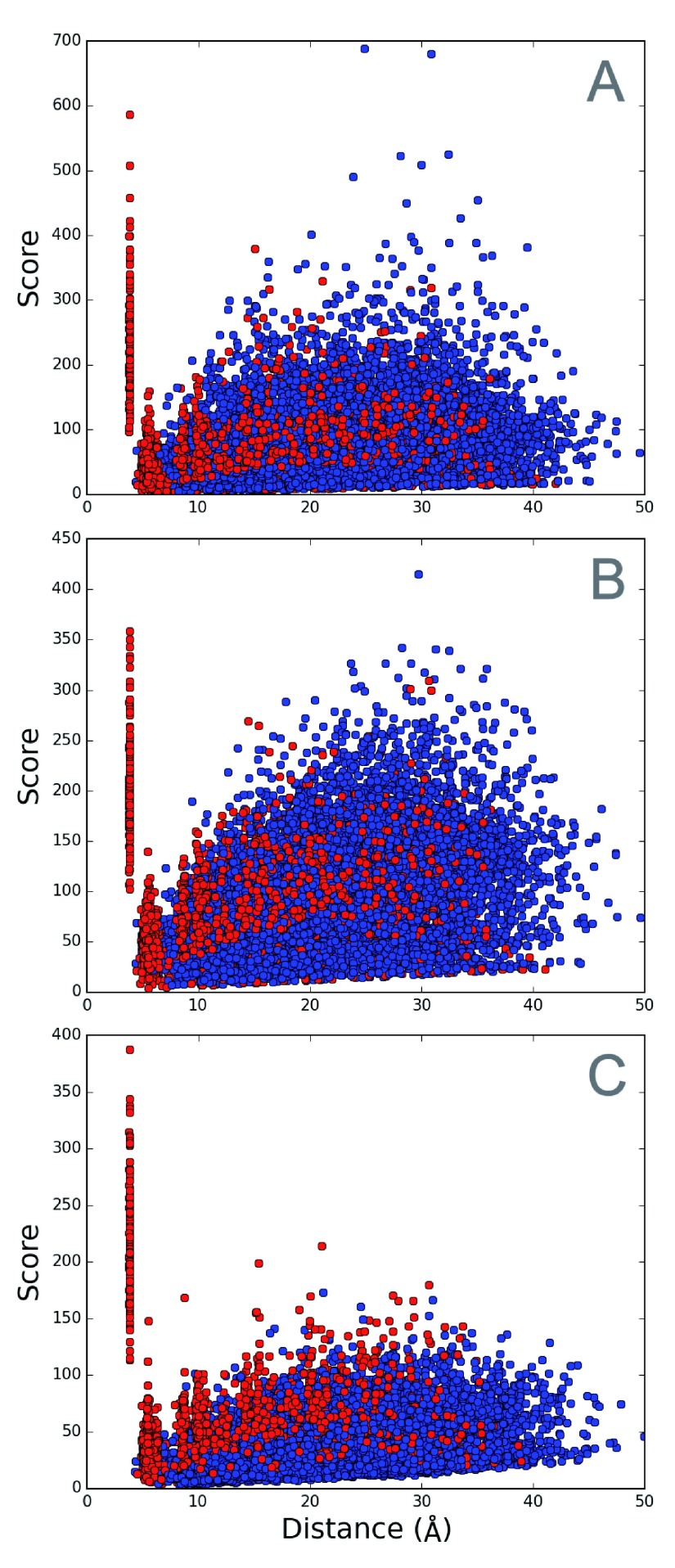
Correlation between score and the average distance for the 14,365 C _α_−C _α_ pairs. **A**. set 1, **B**. set 2, and **C**. set 3. Intrahelical and interhelical C _α_−C _α_ pairs are colored in red and blue, respectively.

**Figure S3.**
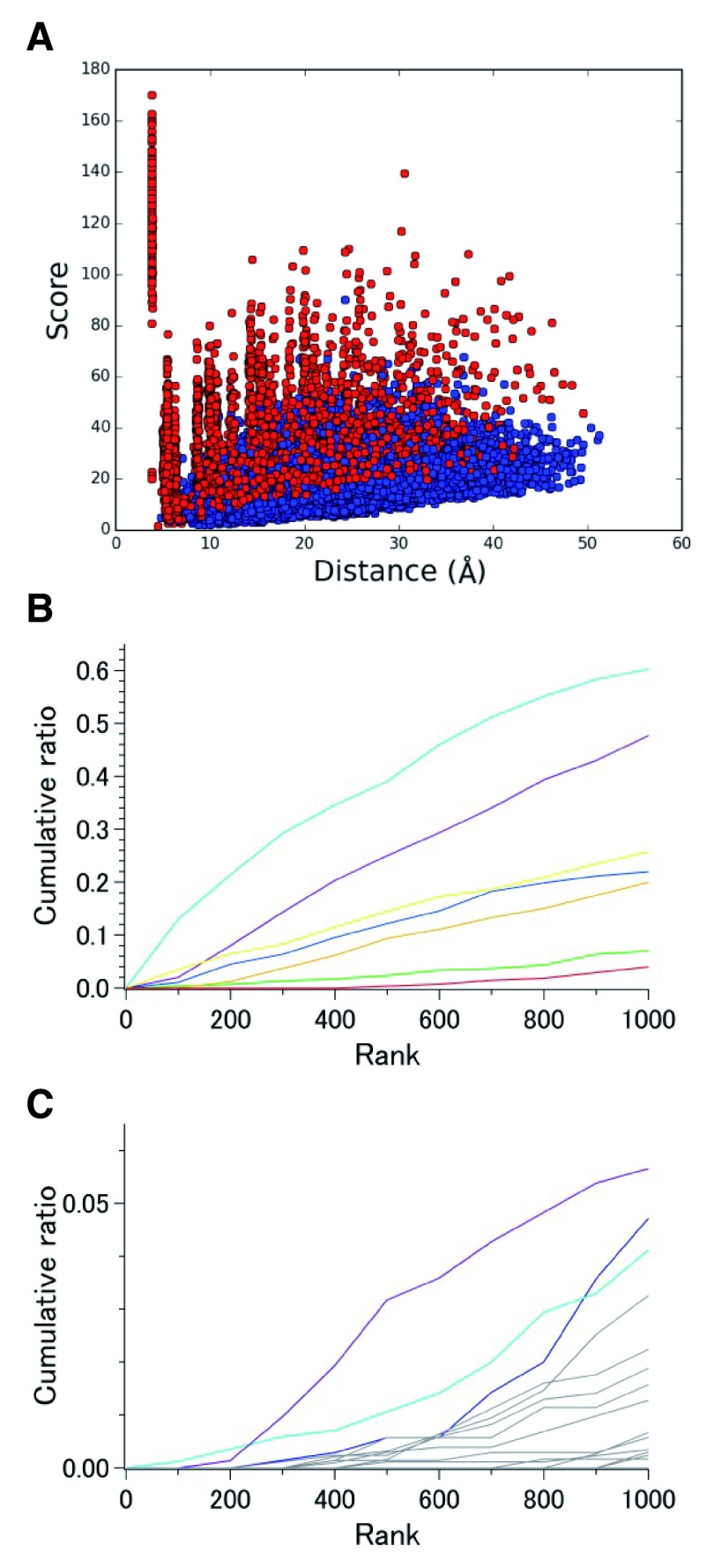
DSA results for all 18,915 C _α_−C _α_ pairs in the updated set of GPCRs containing 23 unique receptor chains. ( **A**) Correlation between score and average distance. Intrahelical and interhelical C _α_−C _α_ pairs are colored in red and blue, respectively. ( **B**) Cumulative ratio of the number of 2,661 intrahelical C _α_−C _α_ pairs in the top-ranked 1,000. Coloring of the plots is the same as that used in Figure 3. ( **C**) Cumulative ratio of the number of 16,254 interhelical C _α_−C _α_ pairs in the top-ranked 1,000. Purple, I-VI; cyan, I-III, blue, I-II; gray, others.

DSA results obtained from these 22 chains in set 2 and from 9 chains in set 1 are shown in Figures 2B and 2A, respectively. Scores for C _α_−C _α_ distances estimated by DSA are defined as the inverse of the coefficient of variation ^11^, and should be higher when the variation among chains is smaller. The plot that includes all 14,365 points demonstrates the distribution of scores against the average distances. The overall pattern depicted in these plots is in contrast to a previous report for GPCRs ^11^ and the updated analysis ( Figure S3). In the case of GPCRs of various sequences, populations with high scores are dominated by the contribution from intrahelical pairs, whereas interhelical pairs exhibit high scores in the bR sets. This result for bR shows that interhelical residue pairs exhibit high scores in a set containing very similar chains, and also suggests that external factors such as crystal lattice packing and solvent conditions that possibly affect the structures tend to highlight single helix geometry changes rather than changes in interhelical arrangements. A comparison between the results for set 1 and 2 indicates that high scores are biased toward longer distances for the interhelical pairs in set 2. This may result from the inclusion of highly similar structures ( Table S4) in set 2.

**Figure 3.**
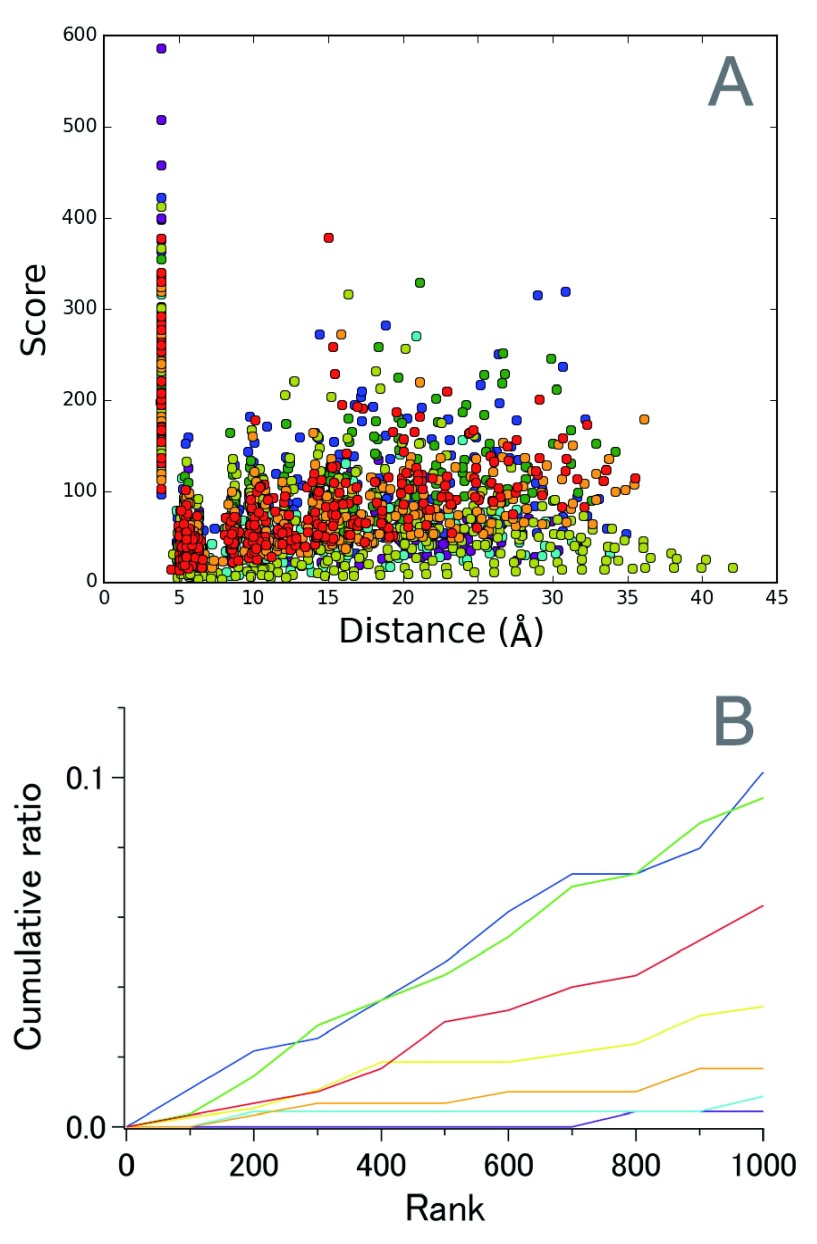
DSA results for 1,992 intrahelical pairs in set 1. **A**. Correlation between score and average distance. **B**. Cumulative ratio of the number of C _α_−C _α_ pairs in the top-ranked 1,000. The pairs are colored as follows; purple, helix A; blue, helix B; cyan, helix C; green, helix D; yellow, helix E; orange, helix F; red, helix G.

**Figure S4.**
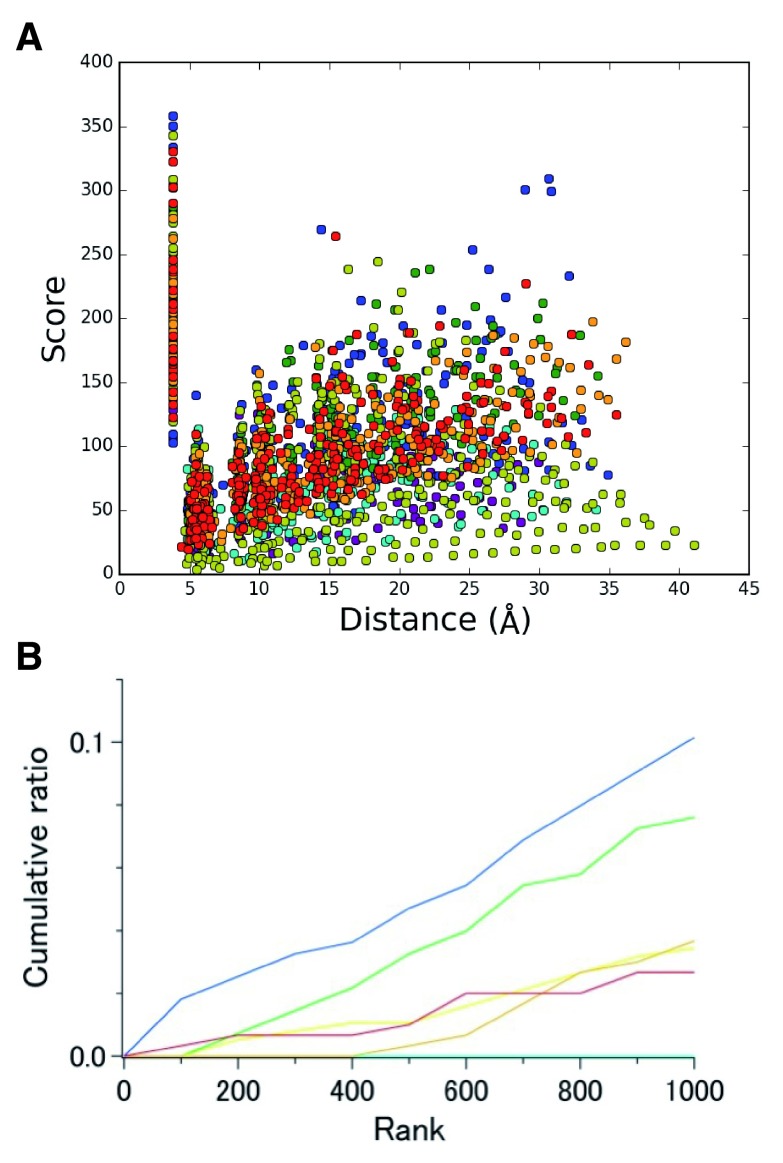
DSA results for 1,992 intrahelical pairs in set 2. ( **A**) Correlation between score and average distance. ( **B**) Cumulative ratio of the number of C _α_−C _α_ pairs in the top-ranked 1,000. The pairs are colored as follows; purple, helix A; blue, helix B; cyan, helix C; green, helix D; yellow, helix E; orange, helix F; red, helix G.

When the intrahelical components were examined in detail, some pairs with high scores were found to originate from helices B and D in both sets 1 ( Figure 3) and 2 ( Figure S4). This finding is more clearly demonstrated by the cumulative numbers (expressed as ratios relative to the total number) of the C _α_ pairs ranked in the top 1,000 ( Figure 3, lower panels). This feature of helices B and D is in contrast to the nearby helices A and C, for which few pairs appear in the top 1,000 ranks. Pairs with the highest scores for helix B were between the residues of inward-facing intracellular region and the residues of lipid-facing extracellular region, and for helix D involved the cytoplasmic (amino) terminal residues. The implications of these findings will be discussed later.

**Figure S5.**
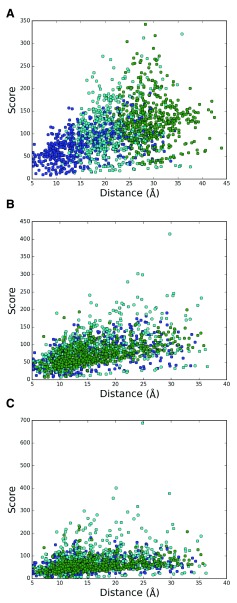
Correlation between score and average distance of a part of the interhelical pairs of bR structures. ( **A**) set 2, blue, A-B; cyan, A-C; green, A-D. ( **B**) set 2, blue, A-B; cyan, B-C; green, C-D. ( **C**) set 1, blue, A-B; cyan, B-C; green, C-D.

To examine whether useful information can be obtained by analyzing interhelical components, we first checked the distance dependence of scores. In principle, this is easily done when a comparison is made among the helix pairs such as A-B, A-C, and A-D, the latter of which contains longer-distance pairs. As shown in Figure S5A, it is apparent that A-D pairs tended to exhibit higher scores than A-B, and A-C in the case of set 2. Therefore, a baseline correction or comparison of scores within a limited range of distances should be made when evaluating the pairs with high scores in such cases. When we compare helix pairs of similar distances, like A-B, B-C, and C-D, however, such distance dependence was not obvious ( Figure S5B) and some remarkably high scores are found for B-C pairs. Importantly, more conserved B-C pairs were discernible even when the number of chains considered was limited to 9 as in set 1 ( Figure S5C), which contains chains of either different space groups or research groups who solved the structure ( Table S2). The high score B-C pairs were between the residues of lipid-facing extracellular region in helix B and the residues of intracellular region in helix C. The former is consistent with the above-mentioned results for intrahelical pairs and the latter contains a cluster of leucines and Asp96 (3.64) which is implicated to be important for proton pumping function. From these results, we suggest that just under 10 chains of very similar structures can provide statistically significant information regarding the relatively insensitive intramolecular spacing of a protein against external forces.

### Distance analysis of MR structures

The results for wild-type bR ground-state chains demonstrate how DSA scores represent intramolecular distance changes against environmental factors even in the absence of sequence variation. On the other hand, analysis of set 3, which contains 13 chains of unique MR sequences, is expected to clarify the part of 7TM that is the most structurally conserved among the evolutionally related proteins. Although the number of available chains is fewer than the previously examined sets of GPCRs, we found that the overall pattern observed for all 14,365 pairs ( Figure 2C) was more similar to that of 18,915 pairs of GPCRs ( Figure S3A) than that of 14,365 pairs of dark-state wild-type bR ( Figure 2A, 2B). This observation confirms that the contribution of interhelical pairs to the high-score population becomes insignificant when sequence variation is involved.

**Figure 4.**
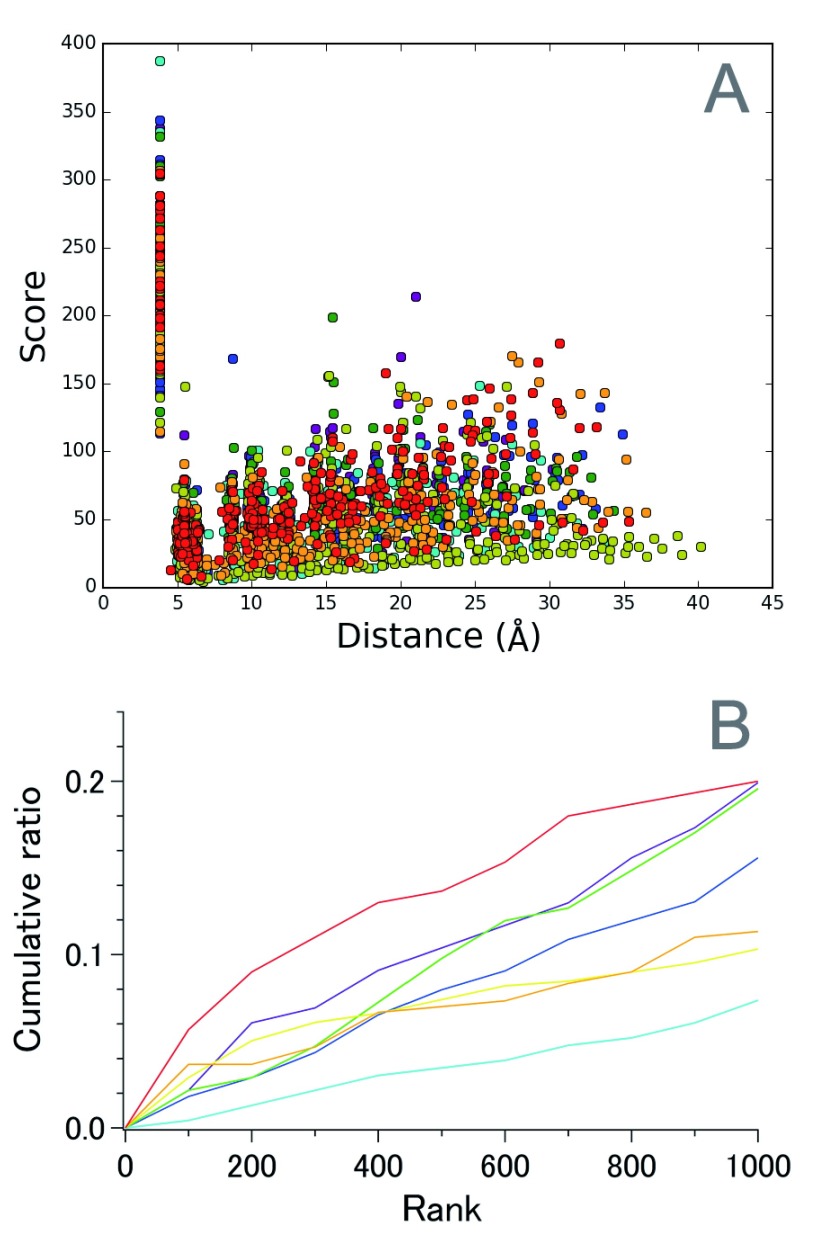
DSA results for 1,992 intrahelical pairs in set 3. **A**. Correlation between score and average distance. **B**. Cumulative ratio of the number of C _α_−C _α_ pairs in the top-ranked 1,000. Coloring of the plots is the same as that in Figure 3.

The most prominent intrahelical pairs with high scores were from helix G ( Figure 4), to which retinal chromophore is attached. This finding is reasonable if we consider that all 13 proteins require retinal binding to a specific site, Lys(7.50), for their function as photoreceptors. Interestingly, the middle of this helix contains a π bulge within which Lys(7.50) resides ( Table S5). Thus, it appears that intrahelical distance conservation is not dependent on whether a helix assumes a regular geometry or not. This finding adds an important revision to the previous view that the remarkably high score observed for helix III in the 7TM bundle of GPCRs might be partly explained by its regular helical structure ^11^.

It should also be noted that helix C appeared to be the most variable among the seven helices of MRs ( Figure 4B). This was rather unexpected taking into account the fact that this helix contains highly conserved residues in addition to Arg(3.50), including Tyr(3.51), Trp(3.54), and Pro(3.59) ( Table S1), and these residue types are completely conserved in 13 chains examined here by DSA ( Figure S2). These observations suggest that intramolecular distance conservation among a set of evolutionally related proteins cannot always be inferred from the degree of sequence conservation. The structurally variable nature of helix C among 13 MRs may be in line with the finding that it does not contain many high score pairs in top 1000 ranks of dark-state wild-type bR sets ( Figure 3). Another possible explanation for low scores of the pairs in helix C appears to be a substantial displacement in the backbone position in 2 halorhodopsin chains around the 3.53 position (Asp in most MRs, and Thr in 2 hRs), whereas an Asp to Asn mutation at this position in the structure of blue-absorbing proteorhodopsin (D97N) does not affect the structure of this region significantly.

**Figure 5.**
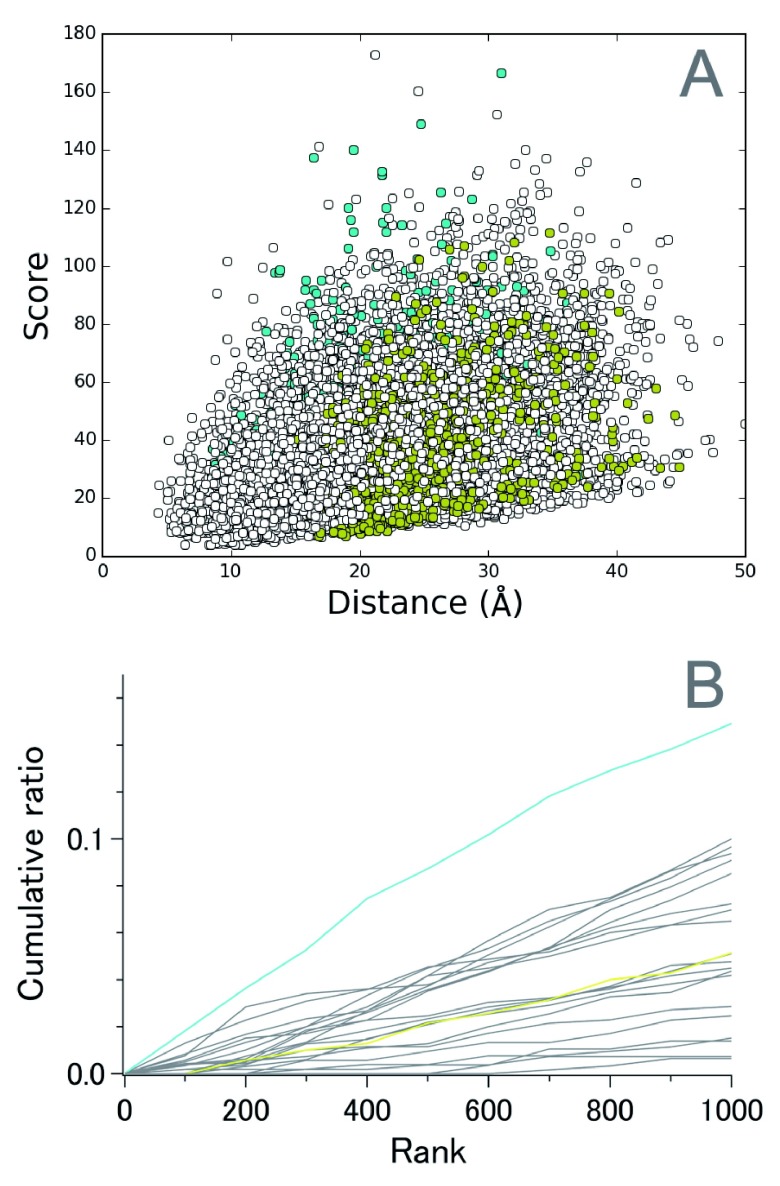
DSA results on 12,373 interhelical pairs in set 3. **A**. Correlation between score and the average distance. **B**. Cumulative ratio of the number of C _α_−C _α_ pairs in the top-ranked 1,000. The pairs are colored as follows; cyan, C-G; yellow, E-G; gray, others.

**Figure 6.**
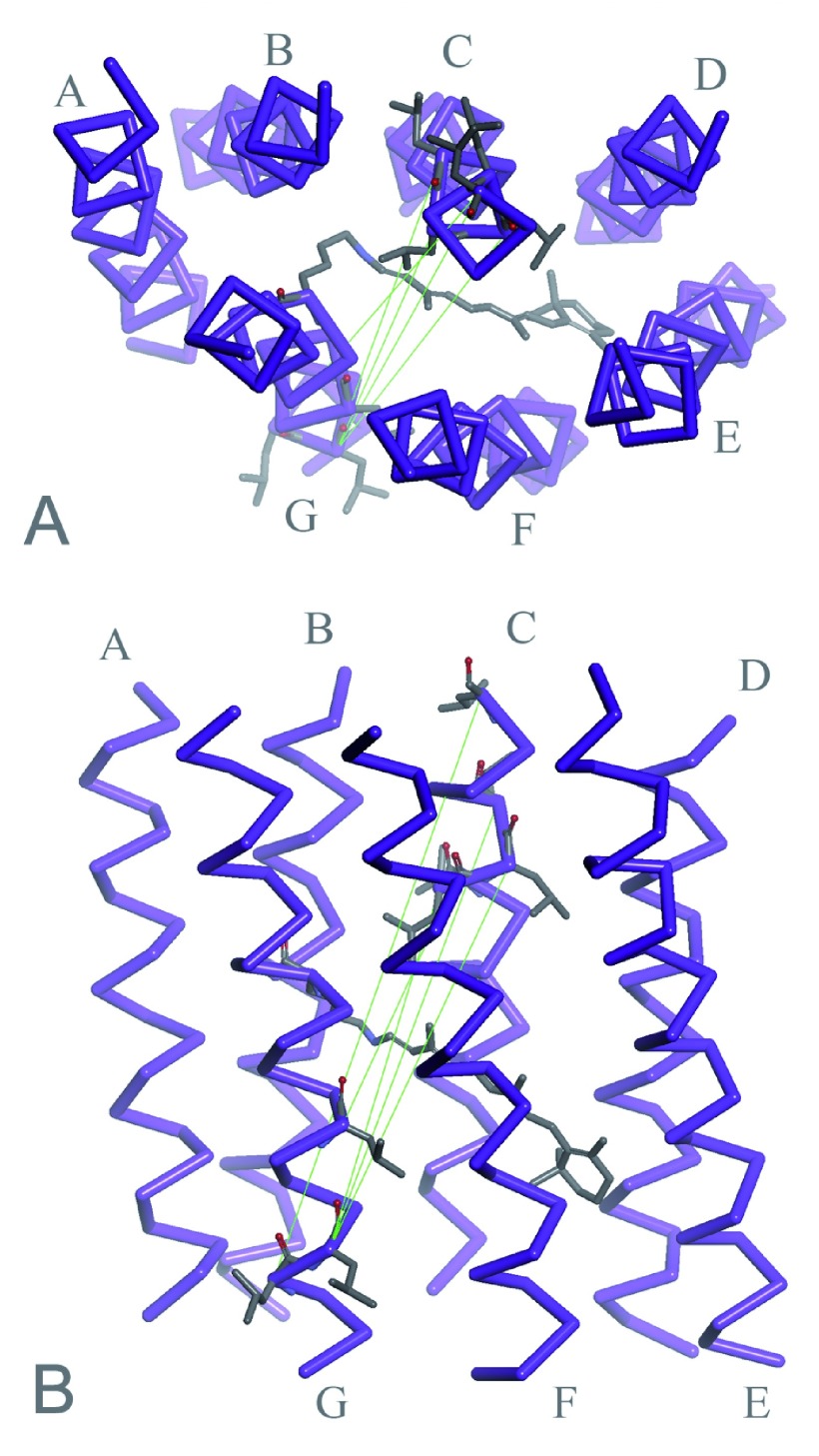
Graphical representation of conserved interhelical distances in set 3 including 13 unique bundles from all MRs with known structure. **A**. Top view from the cytoplasmic side. **B**. Side view from helices F and G. The pairs between helices C and G with high scores are connected by green lines drawn on chain A of 1PY6 (bR). A retinal chromophore attached to Lys(7.50) is also shown in the center.

As Figure 2C demonstrates, there was little distance dependency among the interhelical pairs in set 3; therefore, we examined the pairs in detail and noticed that a remarkable contribution to the high scores was attributed to the pairs between helices C and G ( Figure 5, cyan). Since other interhelical pairs did not exhibit significant features, only E-G pairs are colored in yellow as a reference. The pairs with the highest scores involved the residues on the intracellular side of helix C and extracellular side of helix G, as shown in Figure 6. Relatively conserved spacing between these two regions is likely to ensure the binding and Schiff base protonation of all- *trans*-retinal chromophores to the cavity within a 7TM bundle of all MRs of known structure.

## Discussion

### External factors affecting crystal structure

In the present study, we first examined how different crystallization conditions affect the structure of ground-state wild-type bR. We used 22 chains for this purpose, the resolutions of which ranged from 1.8 to 3.5, including 2 chains obtained by cryo-electron microscopy. These structures were solved in different solvent environments and lattice packing. Obvious differences among 22 chains were discernible mainly at the cytoplasmic terminal region of helix E by visual inspection after superimposition ( Figure 1A). This observation appears to explain why pairs with very low scores come mostly from this particular helix ( Figure 3A, yellow). On the other hand, other regions in the 7TM bundle exhibit only moderate deviation, so our quantitative study by DSA is expected to work well for extracting information regarding structural conservation rather than variation.

Our finding that helix B is the most insensitive to external factors may reflect its inherent properties. A previous simulation study on the individual helices of bR suggested that the structures of helices A, B, and E are stable in sodium dodecyl sulfate micelles ^13^. Another possibility is that helix B does not suffer from crystal packing effect. To address this, we examined the molecular arrangement in all 6 space groups. In 5 of the 6 space groups, including native P3 observed by electron microscopy on purple membranes, lateral interactions between helices B and D were found. Therefore, pairs with high scores found in these two helices ( Figure 3) may reflect a stabilization effect owing to crystal lattice contact. Alternatively, inherently stable parts of helices B and D might contribute to the preference of trimeric arrangement for bR by providing suitable intermolecular interactions.

**Figure S6.**
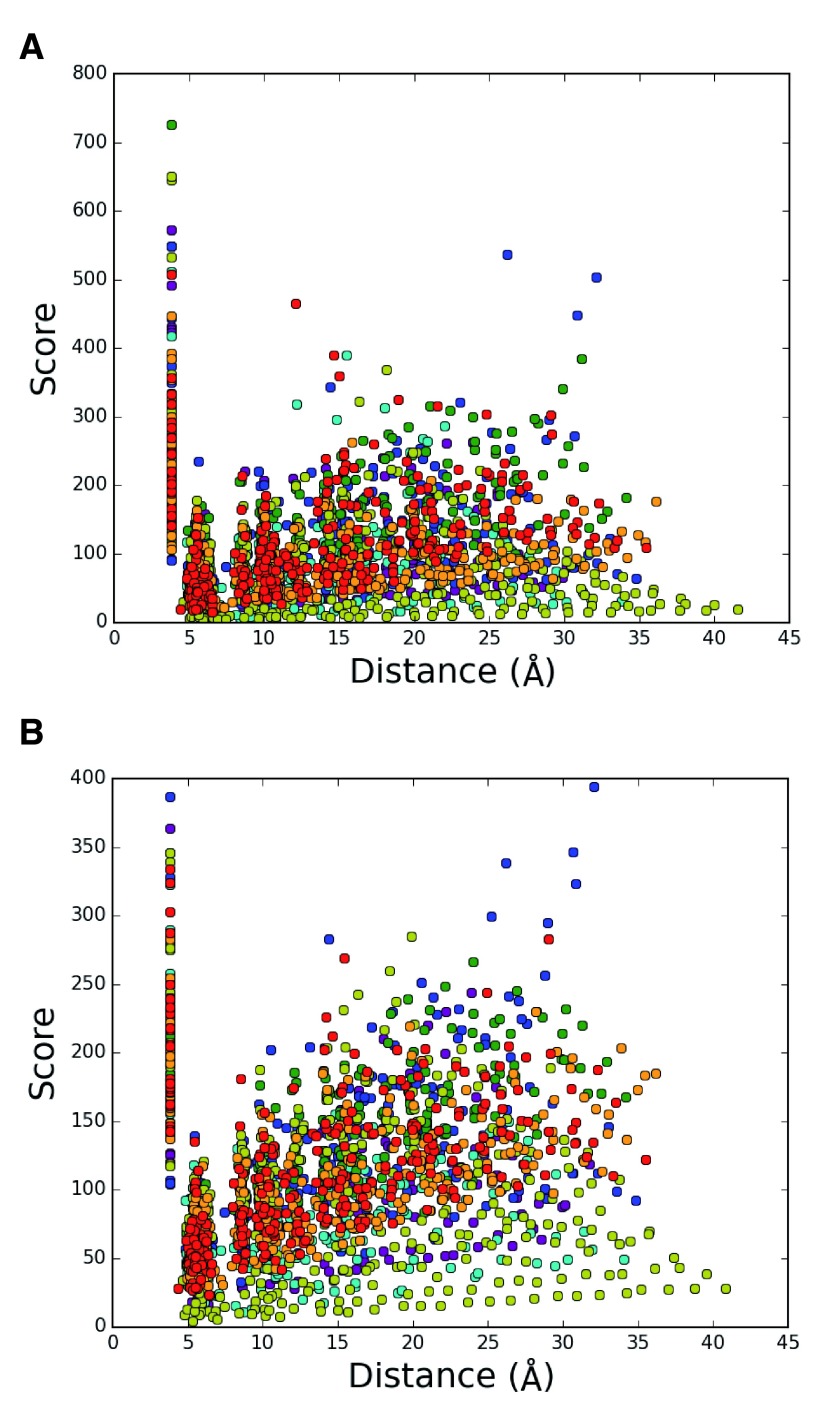
Correlation between score and the average distance for 1,992 intrahelical pairs without a chain obtained from cryo-electron microscopy entry 2AT9 in ( **A**) set 1 and ( **B**) set 2, The pairs are colored as follows; purple, helix A; blue, helix B; cyan, helix C; green, helix D; yellow, helix E; orange, helix F; red, helix G.

Considering that helix C contains a few residues that are important for the proton-pumping function of bR ^14, 15^, such as Asp85 (3.53) and Asp96 (3.64), it may sound curious that this helix does not contribute to pairs with high scores in sets 1 and 2. In fact, removal of a chain that exhibits distinct features can substantially affect the results and result in higher scores for some pairs in helix C ( Figure S6) in set 1 (9 chains) but not in set 2 (22 chains). Therefore, careful examination of each data set is required especially when the number of chains is limited.

### Conservation among MRs

We further performed DSA on the crystallographic models of 13 MRs, the sequences of which vary. The pairwise sequence identity ( Table S3) ranges from 18.2% (between anabaena sensory rhodopsin and blue-absorbing proteorhodopsin) to 88.8% (between archaerhodopsin-1 and 2). This variation was less than that observed among previously analyzed and updated GPCRs ( Table S3). Accordingly, the overall pairwise root-mean-squared deviation was smaller among the 13 MRs (~2.3 Å at most between xanthorhodopsin and blue-absorbing proteorhodopsin) than among GPCRs (~6 Å at most between PAR1 thrombin receptor and CRF1 receptor) ( Table S4) and this is reflected in the relatively higher scores in MRs than GPCRs ( Figure 2C, Figure S3). However, both sets exhibited higher scores for intrahelical residue pairs than interhelical pairs, the latter of which might be more affected by sequence variation.

The high distance conservation between pairs in helices C and G found in the present study suggests that the DSA procedure is useful for detecting structural conditions necessary for common functionality of evolutionally related proteins. Whereas it appears that a slight distance dependency of scores may exist ( Figure 5A), the largest contribution to populations exhibiting high scores for the pairs between helices C and G is not likely explained by such an effect.

In the case of MRs, all members are required to ensure binding of all- *trans*-retinal molecules in a cavity surrounded by 7 TM helices. Whereas helices C and G are in contrast to each other with regard to the degree of intrahelical structural conservation ( Figure 4), our results suggest that a strict condition of spacing between the cytoplasmic terminal region of helix C and the extracellular side of helix G must be fulfilled in all MRs ( Figure 5, Figure 6). Interestingly, retinal Schiff base bound to the side chain of Lys(7.50) resides just in the middle of this conserved spacing ( Figure 6). We suspect that definite structural requirement for MRs, whatever the functions are (pumps, channels, or sensors), would be proper relative positioning of Lys(7.50) and a set of residues from helix C which contribute significantly to holding of the retinal polyene chain and protonation of the Schiff base.

### Implications for the structural biology

Intramolecular distance information from existing crystal structures has long been utilized in the field of structural biology for such purposes as domain recognition ^16^, construction of new models ^17^, and detection of conformational changes ^18^. Although the DSA method might require further improvements, it can be applied in the current form, to the detailed mining of information from larger sets of data than previously examined, and specifically to a number of protein families given that reliable alignments can be obtained. Among the membrane proteins in PDB, the largest category with more than 180 entries is ion channels that transport potassium, sodium, and protons. These proteins function as multi-subunit complexes and exhibit no similarity with any of the 7TM proteins. The second and third-most represented membrane protein families in PDB, MRs and GPCRs studied by DSA, had an advantage in that their alignments were rather straightforward. The present study suggests that around a dozen experimental structures with related and aligned sequences or obtained under distinct conditions can be used to infer statistically significant features of a protein or protein family. From this perspective, a structural archive would be a far more valuable source of information to improve our understanding of biological macromolecules.

## Methods

### Sequence analysis

Microbial rhodopsin sequences were obtained from InterPro ( www.ebi.ac.uk/interpro/) v.48 under the classes archaeal/bacterial/fungal rhodopsin (IPR001425) and archaeal/bacterial/fungal rhodopsin-like (IPR029730). Archaeal proteins did not differ significantly between the two classes, while bacterial and eukaryotic proteins were highly enriched in the IPR029730 class. As the excess bacterial proteins in the IPR029730 class were mostly proteorhodopsins, the sequence set was constructed from the IPR001425 archaeal and bacterial proteins (518 and 298 sequences, respectively) and IPR029730 eukaryotic proteins (651 sequences). A multiple sequence alignment was performed with ClustalW ^19^ implemented in BioEdit 7.2.5 ^20^ for each of the three domains. Based on manual inspection of the results, misaligned or extremely short or long sequences were removed from each domain set. The results for each domain were then merged and an additional alignment was carried out. The distribution of amino acid types at each position was obtained using the Positional Amino Acid Numerical Summary function implemented in BioEdit.

### Distance analysis

Crystallographic models of MRs were obtained from PDB ( www.rcsb.org/pdb/) and classified manually as listed in our web site ( www.gses.jp/7tmsp/) into several groups such as wild-type and mutant bRs, halorhodopsins, and sensory rhodopsins. These PDB entries (accession numbers are as noted in Table S2~ Table S4) were processed to make single polypeptide chains and further truncated to 7TM bundles of 170 residues manually by Discovery Studio Visualizer 3.1 (Accelrys Inc.), ensuring that the alignments for different receptors were correct. The overall pairwise root-mean-squared deviation and correlation coefficient were obtained by Discovery Studio Visualizer 3.1 (Accelrys Inc.) and pca-excel 1.0 (ss-nakano Inc.), respectively. DSA was performed on the C _α_s of the MR bundles as well as 23 GPCRs with unique sequences (19 rhodopsin-like and 4 non rhodopsin-like receptors), following a recently described procedure ^11^. Briefly, the average, standard deviation, and the inverse of coefficient of variation (score) of each C _α_ pair distance were calculated in each of the sets ( Dataset 1). The 7TM bundle of the P2Y12 receptor (PDB ID: 4NTJ) aligned to rhodopsin-like receptors was assumed to lack a residue at the amino terminus of helix VI (6.29). Similarly, the 7TM bundles of the class C mGluR1 (PDB ID: 4OR2) and mGluR5 (PDB ID: 4OO9) receptors were assumed to lack two residues at the carboxyl termini of helix II (2.66 and 2.67) and VI (6.59 and 6.60). The resulting number of C _α_ pairs was 18,915.

Score vs distance plots were prepared with matplotlib ( matplotlib.org/) by implementing in an original python script for DSA ( Dataset 2), and other graphs were drawn using Igor Pro 6.37 (WaveMetrix Inc.). Protein graphics were prepared with either CCP4MG 2.8.1 ^21^ or Discovery Studio Visualizer 3.1 (Accelrys Inc.).

## Data availability

The data referenced by this article are under copyright with the following copyright statement: Copyright: © 2016 Asano M et al.

Data associated with the article are available under the terms of the Creative Commons Zero "No rights reserved" data waiver (CC0 1.0 Public domain dedication). 

*F1000Research*: Dataset 1. Raw data for DSA ( Figure 2– Figure 6, Figure S3– FigureS6), 10.5256/f1000research.7920.d113285 ^22^

*F1000Research*: Dataset 2. Python script for making a score vs distance plot, 10.5256/f1000research.7920.d113889 ^23^
