# Supplementary material for: Sequence and intramolecular distance scoring analyses of microbial rhodopsins
Source: F1000Res. 2016 Apr 6;5:165. Originally published 2016 Feb 12. [Version 2] doi: 10.12688/f1000research.7920.2 (PMC4792210; doi:10.12688/f1000research.7920.2)
Supplement: Peer review history

# Peer review: Referee response for version 2

**DOI:** 10.5256/f1000research.9078.r13276

**Published:** 2016-04-08

**Authors:** Minoru Sugihara

**Affiliations:** 1 Bioinformatics, Meiji Pharmaceutical University, Tokyo, Japan

The authors appropriately corrected the table and figures. Accept it with no changes.

I have read this submission. I believe that I have an appropriate level of expertise to confirm that it is of an acceptable scientific standard.

# Peer review: Referee response for version 1

**DOI:** 10.5256/f1000research.8526.r12405

**Published:** 2016-03-14

**Authors:** Minoru Sugihara

**Affiliations:** 1 Bioinformatics, Meiji Pharmaceutical University, Tokyo, Japan

In this manuscript, Asano *et al.* have applied the own developed method, SDS, to microbial rhodopsins. The basic idea of this scoring is to calculate all Carbon-alpha pairwise distances in each crystal structure and to avoid the ambiguity from the structure-matching.

The manuscript is well presented and the method/results will be of interest to broad readers. I would recommend it for acceptance.

Some minor comments:

Authors propose a new numbering scheme (*.50) from the conservation rate of residues. Adding conservation rates of key residues to the legend of Fig.S1 or Table 1 is useful (one need not to check Table S1). Also in Figure S1 only the residue number “216” of Helix 7 (7.50) is shown and other numbers are missing.

The different numbering in Figure S1 and Figure S2 is a bit confusing. Adding the numbers of the *.50 residue in the bR sequence (27, 57, 82, 122, 152, 182, and 216) at the bottom of the sequencing alignment (Figure S2) might be helpful.

I have read this submission. I believe that I have an appropriate level of expertise to confirm that it is of an acceptable scientific standard.

# Response

**Published:** 2016-03-24

**Authors:** Tetsuji Okada

**Affiliations:** Department of Life Science, Gakushuin University, Japan

Thank you for the critical reading and valuable comments on our DSA (not SDS) method. According to your suggestions, we have revised Table 1, Figures S1 and S2. An accidental error found in the previous Table S1 is also resolved.

# Peer review: Referee response for version 1

**DOI:** 10.5256/f1000research.8526.r12678

**Published:** 2016-03-01

**Authors:** Javier V. Navarro

**Affiliations:** 1 Department of Neuroscience & Cell Biology, University of Texas Medical Branch, Galveston, TX, USA

This article describes a novel structural analysis of microbial photoreceptors by scoring intramolecular distances derived from their high resolution crystal structures. This work could potentially provide the structural foundation to explain the diverse photoreceptor phenotypes, including identifying the structural factors governing their colour tuning, which is still an unresolved problem.

I have read this submission. I believe that I have an appropriate level of expertise to confirm that it is of an acceptable scientific standard.

# Response

**Published:** 2016-03-02

**Authors:** Tetsuji Okada

**Affiliations:** Department of Life Science, Gakushuin University, Japan

We appreciate the positive referee comment. Further results on the MR subfamilies will be discussed in terms of the color tuning mechanism.
